# Electromagnetic Metrology on Concrete and Corrosion*

**DOI:** 10.6028/jres.116.011

**Published:** 2011-06-01

**Authors:** Sung Kim, Jack Surek, James Baker-Jarvis

**Affiliations:** Electromagnetics Division, National Institute of Standards and Technology, Boulder, CO 80305

**Keywords:** corrosion, iron oxide, nondestructive evaluation, permeability, permittivity, reinforced concrete

## Abstract

To augment current methods for the evaluation of reinforcing bar (rebar) corrosion within concrete, we are exploring unique features in the dielectric and magnetic spectra of pure iron oxides and corrosion samples. Any signature needs to be both prominent and consistent in order to identify corrosion within concrete bridge deck or other structures. In order to measure the permittivity and propagation loss through concrete as a function of temperature and humidity, we cut and carefully fitted samples from residential concrete into three different waveguides. We also poured and cured a mortar sample within a waveguide that was later measured after curing 30 days. These measurements were performed from 45 MHz to 12 GHz. Our concrete measurements showed that the coarse granite aggregate that occupied about half the sample volume reduced the electromagnetic propagation loss in comparison to mortar. We also packed ground corrosion samples and commercially available iron-oxide powders into a transmission-line waveguide and found that magnetite and corrosion sample spectra are similar, with a feature between 0.5 GHz and 2 GHz that may prove useful for quantifying corrosion. We also performed reflection (*S*_11_) measurements at various corrosion surfaces and in loose powders from 45 MHz to 50 GHz. These results are a first step towards quantifying rebar corrosion in concrete.

## 1. Introduction

Over the last 40 years, the cost of replacement of large reinforced concrete structures such as bridge decks has been a driver to develop new and reliable nondestructive methods to quantify the degree of corrosion on embedded rebar. Electromagnetic measurements such as ground penetrating radar (GPR), induction, thermography, and direct-current (DC) electrochemical impedance have been developed, and in some cases used to assess rebar corrosion, but none of these methods quantify corrosion in early stages when remediation and monitoring are useful. At the National Institute of Standards and Technology (NIST), a focused multidisciplinary research and development effort is underway to investigate and extend the use of electromagnetic (EM) waves from 45 Megahertz (MHz) to 3 Terahertz (THz) to characterize and quantify iron-oxide corrosion. This paper presents measurements from 45 MHz to 12 Gigahertz (GHz) on corrosion and concrete samples because this frequency range is most relevant for remote sensing of rebar corrosion in bridge decks. The goal is to find one or more unique radio-frequency (RF) signatures of corrosion that can be quantified *in situ*. Generally, this is a search for spectra related to magnetic absorptions in one or more iron oxides associated with steel rebar corrosion.

To characterize corrosion and concrete, we use a vector network analyzer (VNA) to acquire S-parameters from samples placed in specialized metal conduit waveguides. S-parameters are voltages normalized to the transmission-line impedance that are normalized to the normalized source voltage amplitude and phase at a reference plane “port” chosen here at the sample boundaries. *S*_11_ is the normalized voltage ratio reflected from the front surface of a sample from port 1 and *S*_21_ is the normalized voltage ratio transmitted through the sample of a signal from port 1. These S-parameters together with the sample length are used to compute the dielectric permittivity, magnetic permeability and loss characteristics that we report in the figures. We use waveguides with and without a center conductor to support a propagating wave. A hollow conducting cylinder with a center conductor is a coaxial transmission line that supports a transverse electromagnetic wave (TEM). Used with a VNA, a coaxial transmission line operates from tens of megahertz up to a maximum frequency where the next higher mode above TEM begins to propagate in the line. For the coaxial transmission line measurements we packed a 7 mm air line with iron oxide powders. Here 7 mm air line refers to a transmission line with a cylindrical inner conductor of 7 mm diameter that has a center conductor sized to make the line impedance (ratio of voltage to current) 50 ohms with air as the intervening dielectric. Similarly, we tested concrete samples by machining them to fit into a 77 mm air line, as well as S-band and X-band waveguides. The latter two are standard rectangular waveguides. A 7 mm air line operates up to 26 GHz with air as its dielectric, but has a practical limit of 12 GHz when loaded with iron oxide samples. S-band and X-band waveguides operate from 2.60 GHz to 3.95 GHz and 8.2 GHz to 12.4 GHz, respectively, when filled by air, but again these limits of operation are lowered in the section of waveguide loaded with a concrete or mortar sample. We also perform some *in situ* measurements by placing the cut off, i.e., electrically “open”, end of a 1 mm diameter polytetrafluorothylene (PTFE)-filled rigid coaxial transmission line within corrosion powders or against corrosion surfaces. In this case only *S*_11_ parameters can be acquired and are reported directly in Figs. 11 through 14.

Rebar closest to the surface in a bridge deck is embedded below 4 cm to 8 cm of concrete [1]. In most cases, the primary mechanism that initiates rebar corrosion in bridge decks is chloride ion depassivation of the thin FeO scale that forms on the rebar surface at the time of manufacture. Water, oxygen and chloride ions episodically diffuse through the concrete cover layer with seasonal changes and as road salts are applied for de-icing. These gradually break down the rebar iron, but if the concrete is constructed well then this degradation is very slow, thus allowing many years of service. A failure criterion for reinforced concrete structures has been set at 25 % or more degradation of the rebar cross section due to corrosion [2]. As rebar corrodes inside concrete, iron atoms leave the steel surface and are incorporated into iron-bearing oxides. Because these oxides occupy several times more volume than the originating iron, their accretion eventually causes radial cracks in the concrete around the bar. The critical amount of corrosion to initiate radial cracking in the concrete surrounding a rebar element has been estimated at 30 μm to 272 μm in specimens with localized spots of corrosion, and 3 μm to 74 μm where the corrosion is more uniform [3]. Rebar surface corrosion from 15 μm to 50 μm commonly causes the first visible radial cracks to appear in the cross section of a test specimen. Corrosion growth accelerates with crack growth and eventually the cracks reach the deck surface, strongly indicating the need for replacement or repair. Therefore, nondestructive evaluation (NDE) tools to quantify the corrosion developing on embedded rebar well before significant cracking would allow structural health monitoring and timely repairs to extend bridge life.

Ferromagnets such as iron have spins densely packed in the crystal lattice, and as a result these spins experience positive Heisenberg exchange as a coherent energetic interaction that favors their alignment [4–5]. Additionally, strong magnetic dipolar fields are generated by this alignment that can cause the properties of a ferromagnetic sample to be strongly influenced by its shape and size. The iron oxides that form as corrosion on rebar and other steel elements are mixtures of ferrimagnet crystallites distinguished by differences in stoichiometry and structure. Ferrimagnets have negative Heisenberg exchange, which means that unpaired spins on neighboring iron atoms couple anti-parallel through an intervening oxygen atom [4–5]. An antiferromagnet is a special kind of ferrimagnet where adjacent spin moments completely cancel. Long-range dipolar coupling in ferrimagnets is much weaker by virtue of this pair-wise cancellation, and the dilution of any residual spin moment in these lattices compared to ferromagnets. As a result, we assume that the iron oxide powders tested here have properties close to crystallite behavior they would exhibit in polycrystalline corrosion. More fundamentally crystallites of the 1 μm minimum particle dimension typical for our powders should exhibit essentially bulk ferrimagnetic properties [6–7]. This allows us to proceed with a search for magnetic spectra using relatively pure model systems that is informed by previous scientific investigations on bulk and powder materials. Our open-end coaxial probe measurements at the end of the paper attempt to observe the weak ferromagnetic resonance due to spin canting seen particularly in antiferromagnetic resonance investigations on crystalline α-Fe_2_O_3_, also known as the mineral hematite [6, 8].

The primary EM wave method used to evaluate reinforced concrete structural elements, such as a bridge deck, has been pulsed ground penetrating radar (GPR) imaging [9–15]. Commercial systems are available from 0.5 GHz to 1.5 GHz that can image hidden structure and problems in the deck that involve large contrasts in permittivity. This is accomplished by detecting reflected waves from interfaces at concrete inclusions such as rebar, air and water pockets. If the rebar cross-section is largely corroded away, GPR can reveal this, but such a major problem generally occurs at the end of service life. The resolution [9] of GPR falls far short of that needed to image corrosion layer thickness in the 100 μm range relevant for monitoring structural health.

**Fig. 1:**
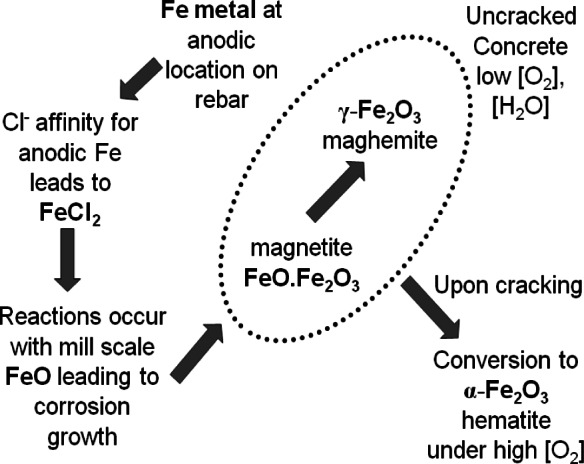
A model of early corrosion growth upon reinforcing bar in concrete, with corrosion growth extending from the FeO oxide scale layer that forms as the metal cools at the steel mill.

There are many possible iron oxides involved in steel corrosion [3, 16], and the combination of oxides that does form is controlled by the local environment. In Fig. 1 we provide a model of the chemical steps involved in rebar corrosion in concrete. Here corrosion formation is limited by the slow episodic diffusion of water, chloride ions and oxygen to the steel-cement interface from the bridge deck surface. As discussed earlier, iron oxides require more volume than the metallic iron precursors [3] and the surrounding concrete medium has little flexibility. For stages where radial cracking is minimal, these conditions generate high pressure which should favor the formation of the densest iron oxides: magnetite, maghemite, and hematite. Indeed, the iron oxides that form as corrosion on rebar embedded in concrete have been found to be largely magnetite (FeO.Fe_2_O_3_) interpenetrated by maghemite (γ-Fe_2_O_3_) along with some hematite (α-Fe_2_O_3_), possibly goethite (α-FeOOH), and other compounds [3, 17–21]. While all iron oxide crystallites in corrosion are ferrimagnets, the iron corrosion states shown in Fig. 1 are antiferromagnetic (FeCl_2_, α-FeOOH, α-Fe_2_O_3_), or ferrimagnetic (γ-Fe_2_O_3_, FeO,.Fe_2_O_3_).

Concrete of low porosity and free from surface microcracks is achieved regularly with standard construction techniques, and therefore many bridge decks have low rebar corrosion growth over many years of service. There is a special need to accurately quantify corrosion as the bridge deck ages to safely and efficiently manage its life to end of service. Both low frequency (< 40 kilohertz (kHz)) inductive and capacitive methods have been used to evaluate rebar corrosion in concrete [22–24]. Thermographic and electrochemical methods have also been tried. None of these provides the quantitative accuracy for monitoring the structural health of rebar.

There is also a strong interest in iron oxide compounds in mineralogy and geophysics [25–30]. Recent measurements similar to ours have been made on hematite and maghemite with the objective of determining the extent of these materials on the planet Mars by the use of radar remote sensing [31] to infer aspects of its geological evolution.

For a spectroscopic approach to be useful, enough spectral information must be obtained above other noise sources. Propagation loss and scattering are serious limitations to the frequency of interrogation. A separate, but related issue is that in order to use magnetic absorbances to quantify iron oxide corrosion, these must be prominent enough with respect to sources of noise in the system, in particular phase noise in test source electronics and noise associated with dipolar losses on the propagating electric field. As a consequence, we tested the RF response of rebar rust constituents in powder form and the propagation losses for concrete representative of the cover layer.

Previous investigators electromagnetically characterized cement-based paste and mortar samples using waveguides from 0.1 to 1 GHz and in some cases 1.5 GHz radar has been used to measure propagation through concrete [11–20]. Our results for concrete cover a broader frequency range that also includes a subset over a range in temperature and humidity common for bridge decks in service. Furthermore, our samples have been cut from a specimen of residential concrete and carefully fitted into S- and X-band waveguide, as well as 77 mm transmission line systems. These samples contained the granite coarse aggregate that is preferred for concrete bridge decks, although with smaller average size. We also poured one cement/sand (mortar) sample into S-band waveguide for direct information on mortar.

## 2. Methods and Materials

In order to study the response of electromagnetic fields applied to both concrete and corrosion independently, we derived dielectric and magnetic properties from S-parameter measurements on representative samples placed in the waveguides noted in the Introduction [32–36] by modeling with Nicolson-Ross-Weir (NRW) type computations [33–34] and the Baker-Jarvis algorithm [37]. We used an HP8510VNA† to measure the S-parameters, which can measure frequency response from 45 MHz through 26.5 GHz.

In this paper, we use notations of materials spectroscopy as $\varepsilon \left(\omega \right)=\varepsilon_{0}\left(\varepsilon_{r}^{\prime }\left(\omega \right)-i\varepsilon_{r}^{\prime \prime }\left(\omega \right)\right)$ and $\mu \left(\omega \right)=\mu_{0}\left(\mu_{r}^{\prime }\left(\omega \right)-i\mu_{r}^{\prime \prime }\left(\omega \right)\right)$, respectively, for the relative dielectric permittivity and magnetic permeability as complex functions of angular frequency *ω*, with *ε*_0_ and *μ*_0_ being the permittivity and permeability of vacuum.

We made measurements on concrete and mortar samples, selectively varying humidity and temperature, in order to study the extent to which these variables modify EM propagation within the range of bridge deck conditions. We poured one sample, a 4:1 mixture of silica sand and type II Portland cement, into a 15 cm long section of S-band waveguide to a thickness of 6.2 cm by capping the bottom end with a polycarbonate plate and letting this cure for one month with the top covered for the first two weeks by another piece of polycarbonate before any testing. For concrete measurements we had samples cut from an old chunk of residential concrete taken from a local concrete company. Samples were cut as follows: (a) a 1.77 cm thick ring to fit into our 77 mm diameter transmission line setup, (b) a 2.5 cm thick rectangular sample with dimensions to fit into an S-band waveguide section, and (c) a 1.25 cm thick sample to fit into an X-band waveguide section. The residential concrete samples had granite aggregate exposed when they were cut, similar to the preferred coarse aggregate in modern bridge decks, but about half the size, roughly from 1 cm to 2 cm in diameter. These samples were honed to shape with diamond tools. However, samples were cut to leave a slight excess in cross-sectional area with respect to the metal waveguide sections and then these surfaces were carefully polished with an abrasive in order to form a tight custom fit between each sample and corresponding waveguide section. This was done to minimize any air-gap capacitances, which is a major cause of inaccuracies in such measurements [38].

Our primary measurements of iron oxides involved packing a 7 mm coaxial air line section with powders ground from harvested corrosion samples and with iron oxide pigment powders. The pigment powders provided a way to isolate spectra of different iron oxide constituents. We obtained hematite, magnetite, and goethite iron oxide pigment powders from Atlantic Equipment Engineers,† with purities of greater than 90 % and particle sizes from 1 μm to 5 μm. Cathay Pigments† supplied a sample of synthetic maghemite powder, normally used in magnetic recording applications. Corrosion samples were harvested as flakes from a bridge girder recently removed from U.S. Highway 34 near Estes Park, CO and from rebar. Rebar samples were obtained from a local contractor both as old recovered rebar that was broken out of concrete, or smaller concrete samples containing corroded rebar found in and around a stack of 10 × 16 foot cut sections of bridge deck from a bridge removed from U.S. Highway 34 near Estes Park, CO. These flakes were ground with mortise and pestle for packing as coarse powders in 7 mm waveguide and later sieved to < 38 μm. Note that the flakes harvested from bridge girder were much larger, darker and denser than the flakes harvested from rebar that was recovered by breaking it out of concrete. We believe that the girder face was covered by the deck surface and that the corrosion from it is more representative of low oxygen, low water environment of early-stage rebar corrosion, while the rebar corrosion here came from flakes that fell off and so are indicative of the higher oxygen, higher water corrosion environment allowed after crack penetration to the deck surface and beyond.

In pure crystals, iron oxides exhibit self-biased ferrimagnetic resonances due to a combination of Heisenberg exchange, crystalline anisotropy, particle size and shape. Because crystalline hematite has a weak ferromagnetic resonance caused by canting of its antiferromagnetically-paired iron spins at 48 GHz and 290 K [8], we anticipate that such resonances may be observable in the polycrystalline iron oxides of corrosion in the range of 30 GHz to 50 GHz and particularly in our antiferromagnetic powders of hematite and goethite. As a first attempt to observe these, we measured the magnitude of reflected wave from the tip of an open coaxial probe known as the Agilent† slim probe. This probe provided *S*_11_ measurements up to 50 GHz and was calibrated to work with our Agilent E8364A precision network analyzer (PNA). In addition to the tip in air, spectra were acquired with this probe inserted into the iron oxide powders as well as ground bridge girder and rebar corrosion samples. The latter two were tested both in the coarse state of previous air line experiments, and after being more finely ground and sieved to particles < 38 μm. The probe also allowed us to measure corrosion surfaces *in situ* and we probed 7 spots on thickly corroded rebar surfaces (~> 200 μm), a black bridge girder corrosion flake, and three spots on thick corrosion transferred from steel to concrete. These open-probe measurements were performed to look for significant “sharp” changes in reflected wave magnitude across this broad frequency range. This, plus the relatively uncontrolled environment of probe tip in contact with corrosion surface, makes these measurements semi-quantitative.

To anticipate “sharp” magnetic resonance absorption features in our ferrimagnetic samples, consider that most low-loss microwave spinel ferrites have linewidths on the order of 30 Oersteds (Oe), which translates to an absorption peak width of 100 MHz. We therefore acquire S-parameter spectra from iron oxide samples at or below this frequency spacing. As a result, we set the frequency spacing of acquired S-parameters to 89 MHz for the corrosion powders, 50 MHz for the iron oxide pigment powders, and 62 MHz for the open coaxial probe measurements at corrosion surfaces and in powders.

Measurement uncertainties are estimated in applicable figures based on the Joint Committee for Guides in Metrology document JCGM 100:2008 [39].

## 3. Measurements

### 3.1 Measurements for Concrete and Cement

**Fig. 2:**
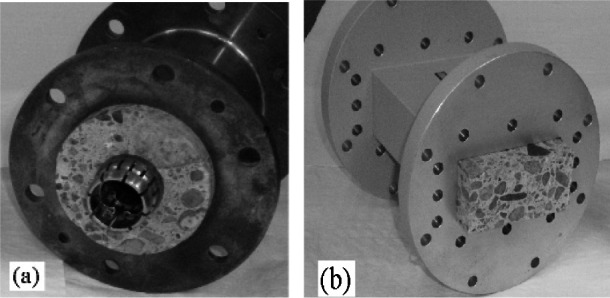
Concrete samples placed in a 77 mm coax transmission line (a) and an S-band waveguide (b).

**Fig. 3:**
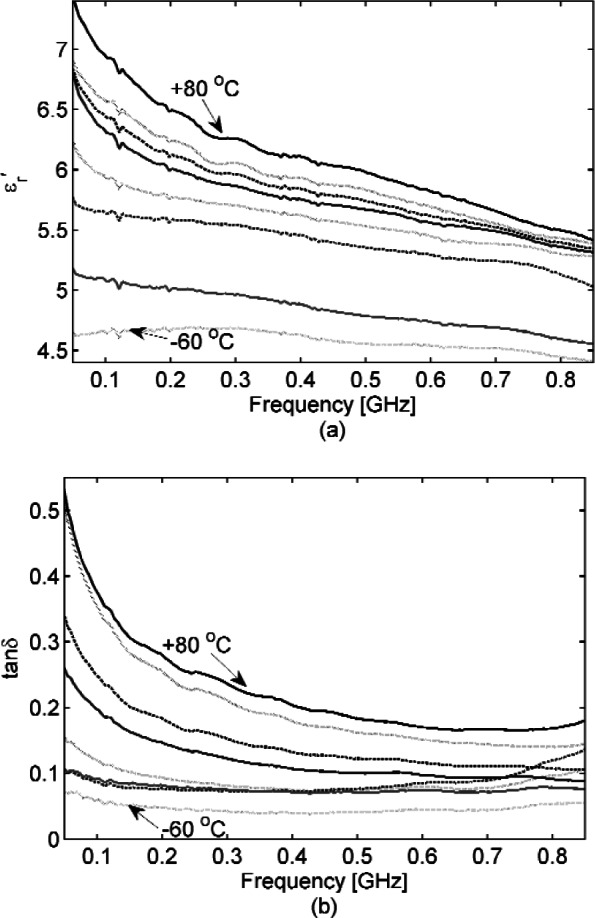
Dielectric permittivity $\varepsilon_{r}^{\prime }$ (a) and loss tangent (b) for concrete with the temperature varied. The 17.7 mm thick sample was inserted into a 77 mm coax fixture. The temperature was increased from –60 °C (bottom curve) to +80 °C (top curve) by 20 °C. The blue solid line is for the dielectric permittivity and loss tangent measured at +22 °C instead of +20 °C. For measured the Type B expanded relative uncertainty is *U = k* u_c_ = 0.2 (*k* = 2) at 0.5 GHz, and for tan *δ* is *U = k* u_c_ = 0.2 *(k* = 2) at 0.05 GHz, where *k* is coverage factor.

**Fig. 4:**
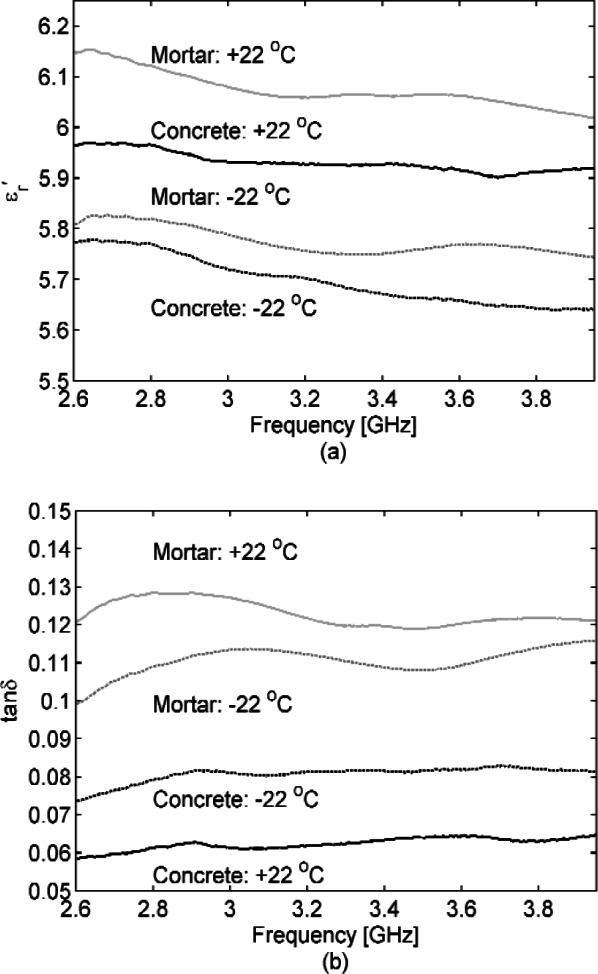
Dielectric permittivity $\varepsilon_{r}^{\prime }$ (a) and loss tangent (b) for 25.4 mm thick concrete and 62.2 mm thick mortar samples inserted/poured into an S-band waveguide section, respectively, with temperatures of + / –22 °C. Less loss is suggested in (b) for concrete at the higher temperature, but actually the loss is not significantly different in terms of our uncertainties. For measured $\varepsilon_{r}^{\prime }$ the Type B expanded relative uncertainty is *U = k* u_c_ = 0.2 (*k* = 2) at 3.3 GHz and for tan *δ* is *U = k* u_c_ = 0.02 (*k* = 2) at 3.3 GHz, where *k* is coverage factor (number of standard deviations).

**Fig. 5:**
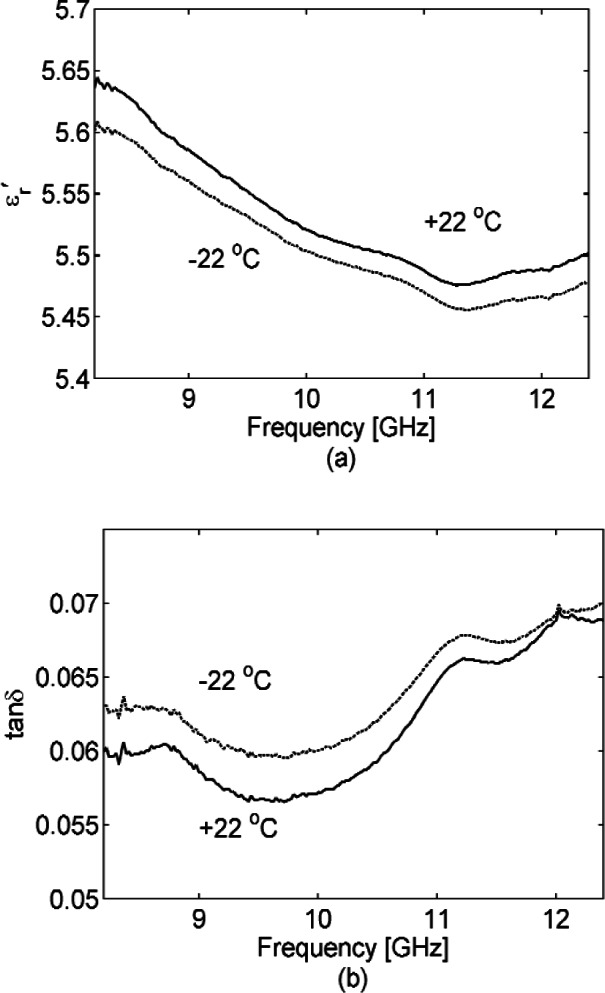
Dielectric permittivity $\varepsilon_{r}^{\prime }$ (a) and loss tangent (b) of a 12.7 mm long concrete sample in X-band waveguide at temperatures of +/−22 °C. As in Fig. 4, the loss tangent for concrete goes down as temperature goes up, but the difference is not significant and so is likely explained by a small systematic error. For measured $\varepsilon_{r}^{\prime }$ the Type B expanded relative uncertainty is *U = k* u_c_ = 0.2 (*k* = 2) at 10 GHz and for tan *δ* is *U = k* u_c_ = 0.02 (*k* = 2) at 10 GHz, where *k* is overage factor.

**Fig. 6:**
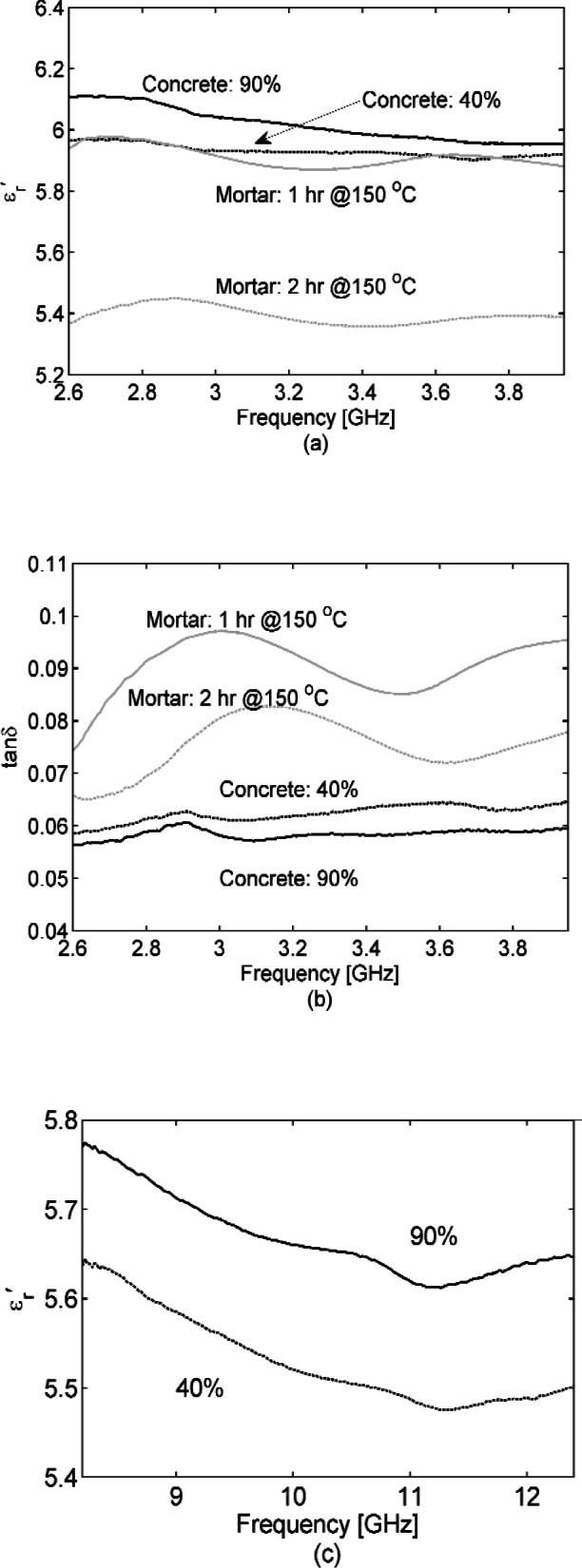

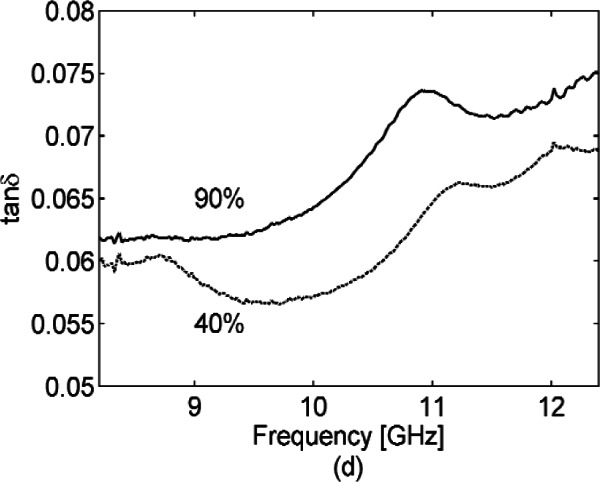
Humidity variation of dielectric permittivity $\varepsilon_{r}^{\prime }$ (a) (c) and loss tangent (b) (d) for concrete. (a) and (b) are in S-band waveguide and (c) and (d) are in X-band waveguide fixture. Results from the mortar sample are also shown in (a) and (b). As-cut, the concrete samples were “dry”— which we interpret here as 40 % humidity, then we soaked them in 90 % humidity for two days in an environmental chamber. By contrast, the mortar sample was measured at about two months into its curing period, so here we heated the sample to 150 °C in a temperature chamber for 1 hour, measured, and then heated the sample to 150 °C for a second hour and measured again. For measured $\varepsilon_{r}^{\prime }$ the Type B expanded relative uncertainty is *U = k* u_c_ = 0.2 (*k* = 2) at 10 GHz and fortan *δ* is *U = k* u_c_ = 0.02 (*k* = 2) at 10 GHz, where *k* is coverage factor.

We acquired S-parameters from 45 MHz to 1 GHz in a 77 mm coaxial transmission line, from 2.60 to 3.95 GHz in S-band waveguide, and from 8.2 GHz to 12.4 GHz in an X-band waveguide, for the cut concrete samples specified in Section 2. We also acquired S-parameters for the mortar sample cured in S-band waveguide. Figs. 2(a) and (b) show concrete samples placed in a 77 mm coaxial fixture and S-band waveguide. Figs. 3, 4, and 5 show the concrete permittivity, i.e., the real part $\varepsilon_{r}^{\prime }$ and loss tangent tan *δ* (see Eq. (1) for the definition) in these bands, respectively with results for the mortar sample added to Fig. 4. These figures demonstrate that $\varepsilon_{r}^{\prime }$ and tan *δ* of the concrete decrease as frequency increases, a behavior well known for polar dielectrics [38] such as water. The separate curves in Figs. 3(a) and (b) show the effect of placing the 77 mm coaxial sample in a temperature chamber; $\varepsilon_{r}^{\prime }$ and tan *δ* gradually decrease as the temperature decreases. At our lowest acquisition frequency of 45 MHz, the loss tangent decreases by a factor of two between +22 °C and −20 °C, but this improvement narrows to about 20 % by 500 MHz. By placing samples in a temperature-controlled oven and ordinary freezer to range them from +/−22 °C, the effect of temperature was also studied at S-band and X-band (see Figs. 4 and 5). In Fig. 6 the humidity was varied for S-band and X-band samples at room temperature (+22 °C). As an alternative, humidity was varied for the newly cured (at this point cured for 2 months) mortar sample in S-band waveguide by removing water. We did this by placing the waveguide with sample in a temperature chamber at 150 °C for one hour, acquiring S-parameters, and then placing the waveguide with sample back into the temperature chamber at 150 °C for an additional hour, after which S-parameters were acquired again. Note that our humidity variations are not an attempt to achieve concrete under the driest or wettest extremes, which would require careful sample weighing. We are interested in “wet” and “dry” as well as “cold” and “hot” conditions of actual use and believe that our variations achieve these ranges.

### 3.2 Skin Depth for Concrete and Cement

Field penetration into materials such as concrete may be summarized by skin depth. This is the depth at which the transverse electric and magnetic fields decay to 1/*e* of their amplitudes upon value entering the surface. It can be calculated from the loss tangent and real part of the permittivity of the material. The loss tangent of the concrete or cement is represented by

(1) $$\tan\delta =\frac{\varepsilon_{r}^{\prime \prime }}{\varepsilon_{r}{}^{\prime }}.$$

The skin depth of the concrete or cement is expressed with

(2) $$\sigma_{s}=\frac{1}{\alpha }\left[m\right],$$

where *α* is the attenuation constant, which is given by

(3) $$\alpha =\frac{\omega }{c\sqrt{2}}\sqrt{\varepsilon_{r}\prime \mu_{r}\prime }\sqrt{\sqrt{1+\tan^{2}\delta }-1},$$

where *c* is the speed of light and relative permeability $\mu_{r}^{\prime }$ is set equal to 1 for concrete and mortar.

**Fig. 7:**
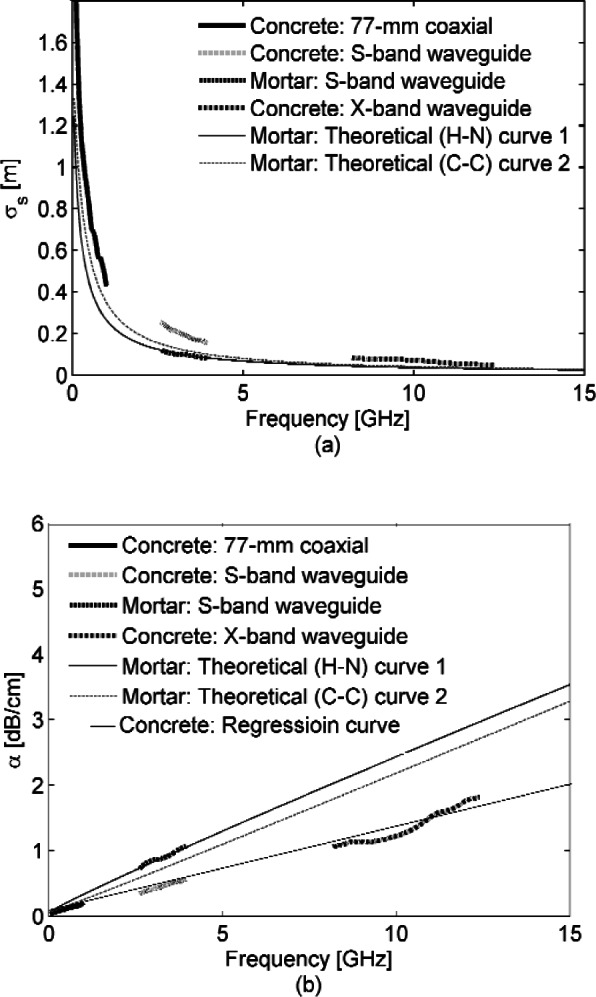
Skin depth (a) and attenuation constant (b) of concrete with data from 77 mm coax, S-band waveguide, and X-band waveguide measurements, as well as measurements from the mortar sample in S-band waveguide plotted against theoretical curves 1 and 2 calculated respectively from Havriliak-Negami and Cole-Cole models based on earlier, more limited measurements.

Figure 7 (a) shows the skin depth of our concrete samples in 77 mm coaxial, S-band, and X-band waveguide sample holders as a function of frequency along with theoretical curves for skin depth generated by fitting to essentially mortar data of previous investigators [40], i.e., these earlier measurements were based on commercial concrete that had the coarse aggregate removed by hand and the mortar that remained was then put into a 14 mm air line waveguide to acquire S-parameters. To calculate skin depths for our concrete samples and the mortar sample, the $\varepsilon_{r}^{\prime }$ and tan *δ* curves given in Sec. 3.1 were used in the above equations, setting *ε*_s_ = 10.5 and *ε*_inf_ = 2 [40]. Typical skin depths from the concrete plots are 56.8, 16.4 and 3.3 cm for 0.8, 3.8 and 12 GHz respectively, while corresponding values for mortar along the Havriliak-Negami fit are 31.3, 8.6 and 3.3 cm. Figure 7 (b) illustrates the attenuation constants for the concrete and mortar samples as a function of frequency. It turns out from these plots that simple regression fits give the dB/cm attenuation loss as 0.143*f* + 0.039 for concrete and 0.238*f* + 0.018 for mortar using this data, taking *f* in GHz. As a further check, consider the 9 dB/cm loss measured by Sato *et al.* from the refractive index of a laboratory concrete sample at 57.5 GHz [41]. At 57.5 GHz the regression equations above estimate propagation loss at 8.3 dB/cm for concrete and 13.7 dB/cm for mortar.

The theoretical curves are based on fits to the Havriliak-Negami and Cole-Cole equations, for details on these see [42]. The skin depth curve for our mortar sample agrees well with these theoretical curves, also based on mortar. The skin depth across all the concrete sample results is about a factor of two longer than mortar skin depth. The simplest explanation is that the coarse aggregate takes up about half of the volume of the concrete and the loss for this granite aggregate is much lower than that of the mortar. Unfortunately, a wide range of coarse aggregate may be used in bridge decks based on regional availability. So this modest advantage with granite may not always be available.

### 3.3 Measurements for Iron Oxide Pigments and Bridge and Rebar Corrosions

For our primary corrosion measurements, iron oxide powders were firmly packed into a 7 mm coaxial transmission line by tamping with a plastic rod and supporting the sample from the bottom with a foam plug. We tested hematite (red), magnetite (black), goethite (yellow), and maghemite (brown) iron oxide powders, as well as corrosion powders ground from flakes harvested from a U.S. Highway 34 bridge girder and from corroded rebar. These measurements were not biased by a DC magnetic field.

**Fig. 8:**
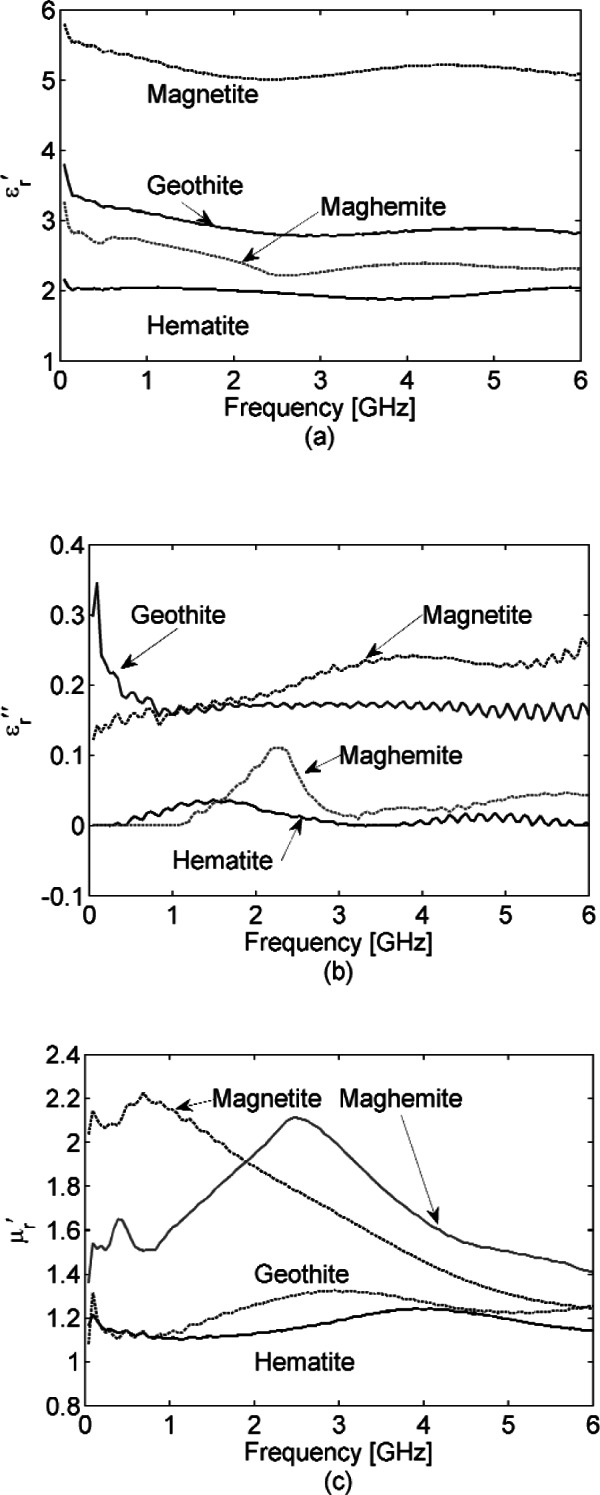

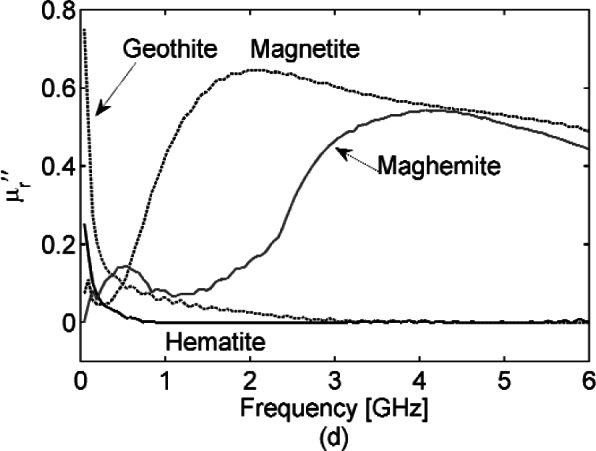
Dielectric permittivity $\varepsilon_{r}^{\prime }$ (a) and $\varepsilon_{r}^{\prime \prime }$ (b) and magnetic permeability $\mu_{r}^{\prime }$ (c) and $\mu_{r}^{\prime \prime }$ (d) of iron oxide powders in the form of 1 μm to 5 μm powders. Each sample was packed in a 7 mm coaxial air line for testing. For measured $\varepsilon_{r}^{\prime }$ and $\mu_{r}^{\prime }$, Type B expanded relative uncertainty is *U = k* u_c_ = 0.1 (*k* = 2) at 3 GHz and for and is *U = k* u_c_ = 0.2 (*k* = 2) at 3 GHz, where *k* is coverage factor.

Figure 8 shows the dielectric permittivity $\varepsilon_{r}^{\prime }$, $\varepsilon_{r}^{\prime \prime }$ (real and imaginary parts in this case for the powders) and magnetic permeability $\mu_{r}^{\prime }$, $\mu_{r}^{\prime \prime }$ spectra for the iron oxide powders. While magnetite, maghemite, hematite and goethite are known to have magnetic properties, the latter two are weak enough that in our measurements they respond as fairly low-loss dielectrics with $\mu_{r}^{\prime }≅1$ and $\mu_{r}^{\prime \prime }≅0$, and their spectroscopic characteristics are constant across our test frequencies. By contrast, magnetite and maghemite show magnetic effects in our measurements ( $\mu_{r}^{\prime }\neq 1$ and $\mu_{r}^{\prime \prime }\neq 0$) and provide unique, if broad, spectral shapes. It is also interesting that magnetite is dispersive, meaning that $\mu_{r}^{\prime }$ and $\mu_{r}^{\prime \prime }$ display peak values respectively at 1 GHz and 2 GHz.

We also performed two X-band electron paramagnetic resonance (EPR) control experiments on the hematite and goethite powders that are not shown. If individual particles of these powders are crystalline on the order of 1 μm then they will have antiferromagnetic coupling similar to that of bulk, but if they are agglomerations of much finer particles, say 19 nm [43], then these particles will lose their antiferromagnetism and exhibit superparamagnetism [6]. To determine whether there was significant superparamagnetism, we filled segments of 22 gauge PTFE tubing with each powder to a length greater than 1 cm. These samples were centered in a TE_102_ EPR cavity using a glass capillary as an alignment sleeve nested in a standard quartz cavity dewar. The conversion time and time constant were set to 80 milliseconds, modulation amplitude to 0.5 mT and gain to 160,000. When continuous-wave X-band EPR was performed on hematite powder using this arrangement, no paramagnetic spectrum was observed with the bias field swept from 5 milliTesla (mT) to 0.99 Tesla (T). Testing goethite with the same setup, we found a small signal near 0.35 T. The center field of this signal shifted lower upon two subsequent acquisitions with the field range reset to 0 T to 0.7 T and then 0 T to 0.25 T. In the latter case, only the high field wing of the spectrum was observable near 0 T. Goethite is known to exhibit a remanent paramagnetic signal due to defects that accounts for these observations [44–45]. These control experiments indicate that our powders have adequate crystallinity to exhibit antiferromagnetism, and crystallite dimensions which approximate the micrometer-range domain size that we assume exists in corrosion.

**Fig. 9:**
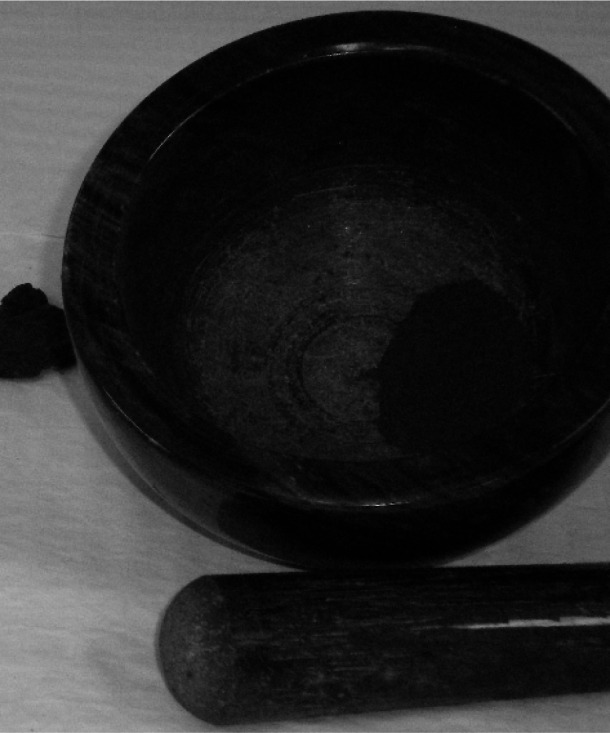
Corrosion powder ground from samples taken from girders.

**Fig. 10:**
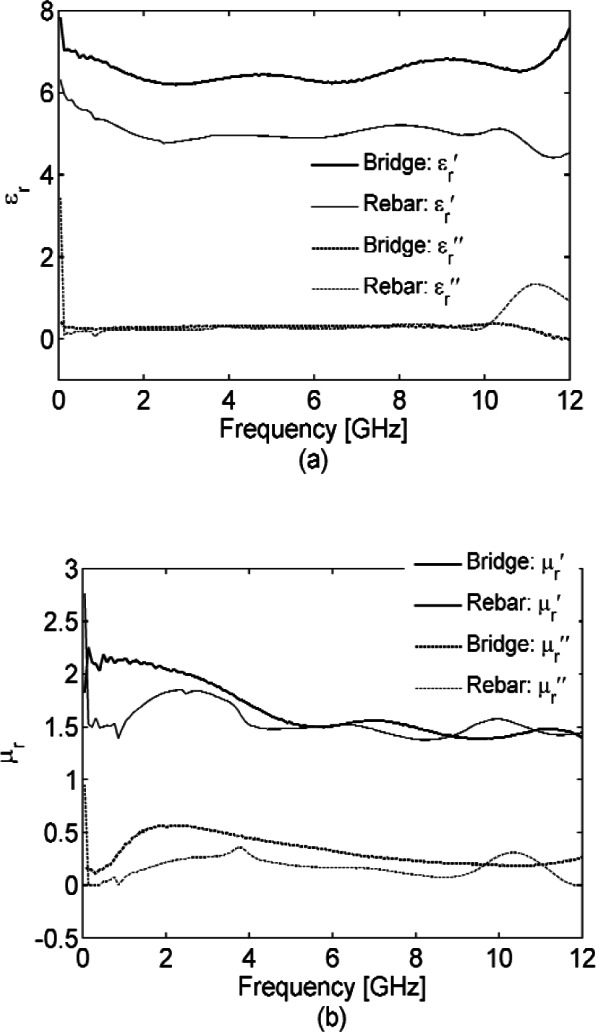
Dielectric permittivity *ε_r_* (a) and magnetic permeability *μ_r_* (b) of the powder samples ground from the bridge girder and rebar corrosion. Each powder was packed in a 7 mm coaxial air line for testing. For measured $\varepsilon_{r}^{\prime }$, $\mu_{r}^{\prime }$, the Type B expanded relative uncertainty is *U = k* u_c_ = 0.2 (*k* = 2) at 6 GHz and for $\varepsilon_{r}^{\prime \prime }$, $\mu_{r}^{\prime \prime }$, is *U = k* u_c_ = 0.2(*k* = 2) at 6 GHz, where *k* is coverage factor.

The mortise and pestle used to grind corrosion samples is pictured in Fig. 9, and Fig. 10 shows the dielectric permittivities and magnetic permeabilities measured for these powders from the bridge girder and rebar corrosion that were packed into the 7 mm transmission line. Their spectral shape and loss (Fig. 10 compared with Figs. 8 (c) and (d) suggest that magnetite is indeed a major constituent of these corrosion samples, with a peak in at 2 GHz.

**Fig. 11:**
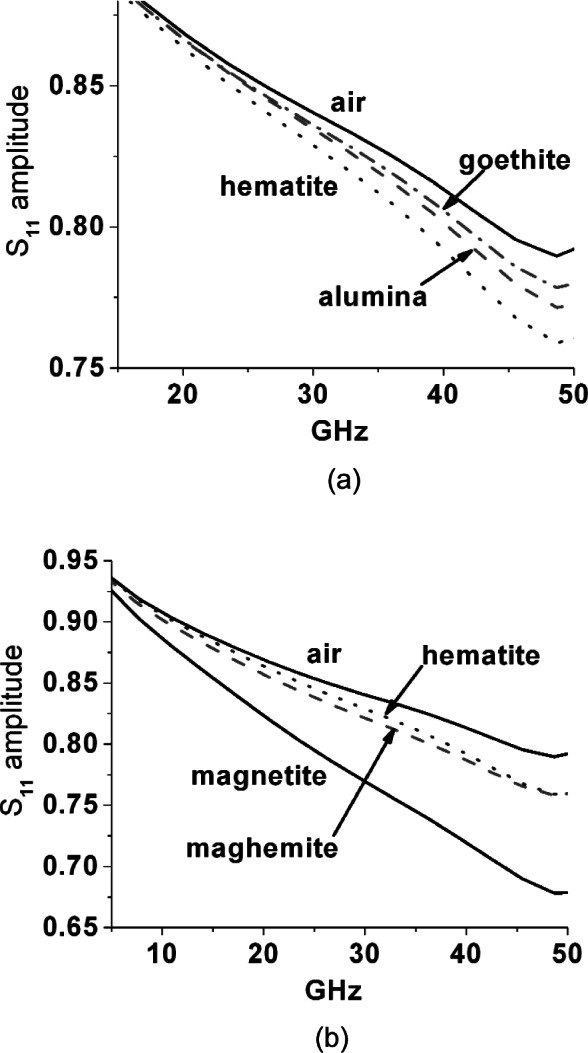
(a) and (b) Linear magnitude of reflected wave from slim probe inserted into 1 μm to 5 μm iron oxide powders-magnetite, hematite, maghemite and goethite. Results for air and 2.5 μm to 5 μm calcined alumina powder shown for comparison.

To check for ferrimagnetic resonances that are self-biased through a combination of Heisenberg exchange, crystalline anisotropy and crystallite size, we used the Agilent† slim probe and report the relative magnitude of reflected wave (*S*_11_) for this probe inserted into iron oxide powders and placed against corrosion surfaces in Figs. 11 through 14. Note that these figures are presented as an ensemble of raw measurements, and as such do not include uncertainty estimates. All measurements with this open coaxial probe exhibit a strong artifact of modulation across the whole frequency band due to resonant reflections across its 20 cm length. To eliminate this we fitted all spectra to sixth order polynomials. For the powders the probe tip was carefully embedded to have a sphere of powder with at least 5 mm radius around it.

In Fig. 11 (a) and (b) the reflection magnitude with the probe embedded in iron oxide powders, in alumina, and in air, are plotted together to show that magnetite again has a distinctly more lossy response, while somewhat surprisingly, goethite appears to be a slightly less lossy dielectric than alumina. There is a subtle downward curvature in the hematite reflection magnitude spectrum to meet the maghemite curve beyond about 45 GHz. In unbiased and relatively large crystalline hematite samples there is a weak ferromagnetic resonance due to canting of the anti-paired spins at 48 GHz at 290 K [8] that could be associated with this. However, the alumina powder with similar particle size shows a similar subtle trend downward in this range.

**Fig. 12:**
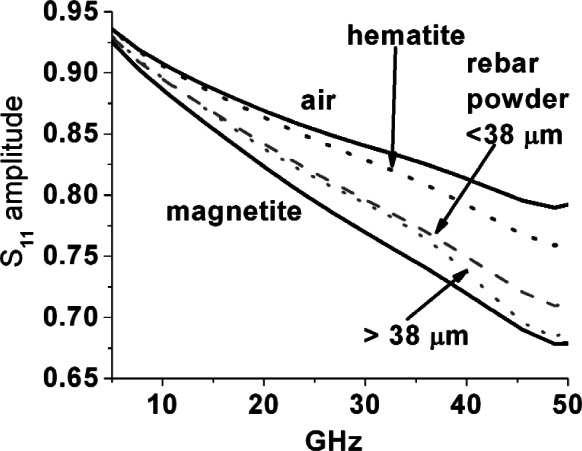
Linear magnitude of reflected waves for slim probe inserted into rebar corrosion powders, one case screened to less than <38 μm in size and the other with particles ranging from 38 μm to ~500 μm in size. Results for probe in air and inserted into hematite and magnetite powders are shown for comparison.

**Fig. 13:**
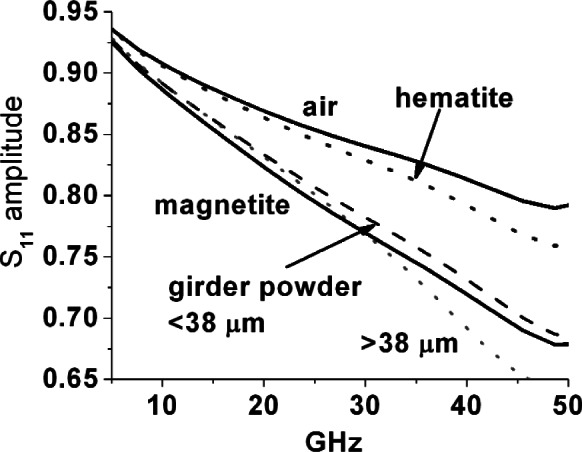
Linear magnitude of reflected wave for slim probe inserted into bridge girder corrosion powders, one case screened to <38 μm and the other with particles ranging from 38 μm to ~500 μmm. Results in air and in hematite and magnetite powders are shown for comparison.

In Figs. 12 and 13, reflection magnitude spectra from corrosion powders ground from rebar and bridge girder samples, respectively, are shown along with the curves for air, hematite and magnetite from Fig. 11 repeated for comparison. Note that two powder distributions were measured in each case; rough powders with particles up to ~500 μm in size that were already used in the loaded 7 mm air line measurements of Fig. 10, where relative permeability and permittivity were extracted, as well as powders that were further ground and sieved to particle sizes of less than 38 μm. The results suggest first that magnetite is indeed a significant fraction of the corrosion samples, because the spectral curves are closest to this powder curve. Secondly, the coarse fractions do show features distinct from the more finely ground fractions, which are a mixed bag—a broad peak of higher-than-monotonic reflection magnitude from 28 GHz to 42 GHz for coarse rebar corrosion, while coarse bridge girder corrosion appears to go lower-than-monotonic past about 30 GHz—the former suggesting incrementally less, and the latter more, loss in these ranges.

**Fig. 14:**
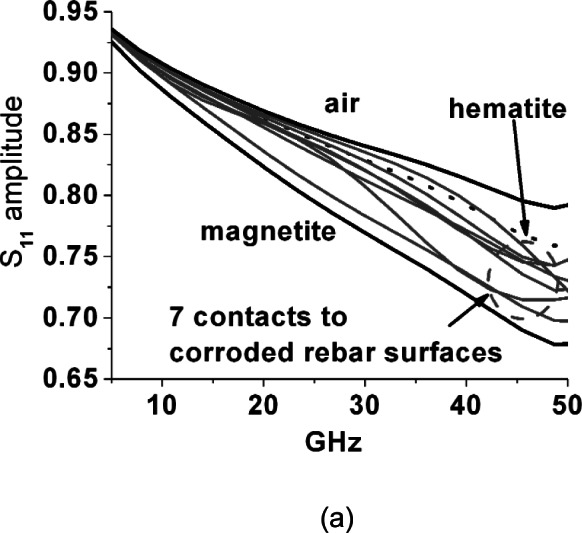

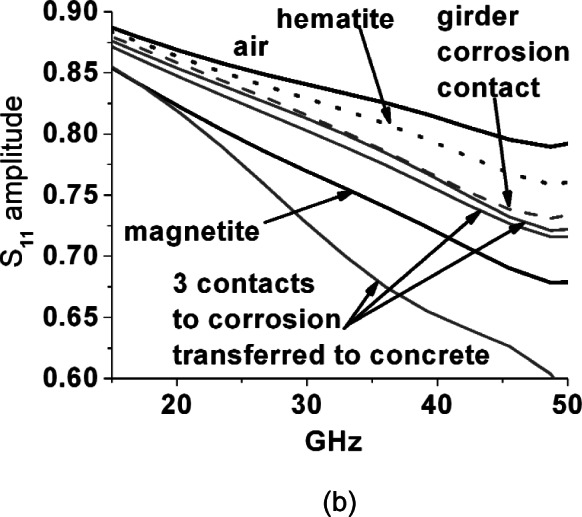
Linear magnitude of reflected wave from slim probe placed against thick iron oxide corrosions at (a) seven rebar surfaces-all clustered between magnetite and hematite powder results shown for comparison and (b) three surfaces where substantial corrosion transferred from rebar to concrete, as well as a dense rust patch taken from a girder that supported the deck of a bridge along U.S. 34 were probed. One corrosion transfer case suggests much higher loss. The results for the probe in air, hematite and magnetite are again repeated for comparison.

Finally, in Fig. 14 (a) and (b) *in-situ* measurements were made by probing corrosion surfaces with the Agilent slim probe. Seven of these measurements were made at the surface of substantially corroded rebar samples, one at a black rust flake removed from a bridge girder surface and three against rebar corrosion thickly transferred to surrounding concrete. These measurements depend on the quality of the temporary probe contact with the corrosion surface, and yet they are remarkable in that virtually all of them have reflection loss magnitudes bracketed by our two lossy iron oxide powders: hematite and magnetite—these powder curves and the curve for air being repeated from Fig. 11 for comparison. These results are also remarkable in that the powders include 30 % to 50 % air. While the corrosion surface looks contiguous under the probe, this confirms significant porosity. In all these *in-situ* measurements no consistently prominent, i.e., “sharp”, spectral absorption features of magnetic resonance up to 50 GHz are evident. One case of corrosion that was completely transferred to the surrounding concrete is a strong outlier with significantly larger absorption across the frequency range, but for two other cases the *S*_11_ curves are well within the range bracketed by the *S*_11_ spectra for magnetite and hematite.

## 4. Discussion and Conclusion

We have performed spectroscopic measurements on concrete, mortar, 90+ % pure iron oxide powders, and iron oxide corrosion samples ground from bridge girder and rebar flake corrosion samples, in addition to *in-situ* broadband measurements of corrosion surfaces. We performed these separate studies to provide measured data from which to estimate signal loss for electromagnetic waves propagating to the corrosion surface of embedded rebar and to determine any spectral features unique to this corrosion that could be leveraged to quantify its extent.

Our concrete measurements agree with work of previous investigators in both the presence and absence of granite coarse aggregate. They suggest that granite aggregate reduces propagation loss by a factor of 2, roughly the amount of volume filled. Our spectroscopy on concrete (and mortar) provides information up to 12.4 GHz, covering more frequencies, and/or extending to higher frequencies, than previous investigations [41, 46–51]. Furthermore, most of our measurements were made on samples cut from concrete that previously was in actual service. These data, along with the mortar data from previous investigators, follow closely the theory of Havriliak-Negami and Cole-Cole, such that skin depth may be theoretically extrapolated/interpolated for frequencies outside of range—thus the real part of the permittivity and loss decrease with frequency. There are two caveats for our concrete data. Coarse aggregate will start to show scattering loss when its average size is greater than one-quarter wavelength of the electromagnetic waves propagating through the sample and the degree of this scattering will depend on how closely the refractive index between aggregate and mortar match at frequencies above this limit. Secondly, the coarse aggregate used in reinforced concrete structures varies with regional availability. Propagation loss and scattering effects in concrete with limestone or other coarse aggregate will likely differ from the results we obtained here for granite. We estimate +/–30 % at this point and plan to extend these measurements.

We found that while permittivity and loss in concrete decrease as the temperature is decreased, the loss decreases by only about a factor of 2 at 45 MHz between + and –22 °C, and this advantage for wave propagation quickly shrinks as frequency increases. So unlike bulk water, which undergoes a drastic reduction in propagation loss in the microwave range when it freezes to ice crystals, the water absorbed in concrete is clearly too segmented and has too many interactions with the polycrystallite surfaces and embedded salts to actually form crystals. Also, at room temperature, we saw only small changes in permittivity and loss in going from 40 % to 90 % humidity, suggesting saturation in the amount of water that the concrete can absorb at this temperature.

We have made fairly extensive measurements of iron oxide powders, and also some semi-quantitative *in-situ* measurements of corrosion surfaces with the Agilent† slim probe version of an open coaxial probe.

Our powder measurements suggest that magnetite is the most significant fraction of early-stage rebar corrosion. The spectral response of ferrimagnets magnetite and maghemite both have characteristic shapes in permeability and loss that may be useful for corrosion quantification in the GHz range while hematite and goethite are lower loss dielectrics with no distinctive spectral shapes in the frequency range we tested. The magnetic permeability and loss of magnetite from 45 MHz to 6 GHz, has a distinct shape, most clearly shown in Fig. 8, and this shape is also reflected in the corrosion samples we ground and tested in this same range, as shown in Fig. 10. This suggests the possibility of using the spectral response of magnetite to quantify corrosion in steel-reinforced concrete structures with emphasis on the frequency range from 0.5 GHz to 2 GHz.

The semi-quantitative exploration of the frequency range up to 50 GHz with the Agilent† slim probe largely confirms out conclusions that real samples of corrosion are significantly loaded by magnetite. While *in-situ* corrosion on surfaces showed more variation, it is remarkable that the results lie between those for hematite/maghemite and magnetite, and in so doing, support the model of interpenetrated maghemite and magnetite iron oxides in corrosion and corrosion surfaces with significant porosity. We saw differences between rough and fine powders in our corrosion samples, and these again suggest that magnetite, having the largest dipolar effects is a controlling influence in the samples. In the case of hematite, where a self-biased ferromagnetic resonance is known for the crystal form at 48 GHz, our powder spectra show a slight indication that may reflect this, but the result is far from conclusive. It should be clear from these *S*_11_ spectra that results can vary widely with specific corrosion surface, and any “sharp” spectral features due to magnetic resonance absorption of anti-aligned spins are not prominent enough to stand out across such a broad frequency range.

## Footnotes

† References to specific hardware, software, and materials in this document are provided for informational purposes only and do not constitute any endorsement by the National Institute of Standards and Technology or imply that their use is the only way to achieve the presented measurements.
